# Applying an implementation science lens to understand physician-level variation in patient length of stay in internal medicine

**DOI:** 10.1186/s12913-025-13304-5

**Published:** 2025-10-03

**Authors:** Diya Srinivasan, Ruoxi Wang, Surain B. Roberts, Lauren Lapointe-Shaw, Terence Tang, Sarah Abigail Birken, Alexandra Harris, Noah M. Ivers, Fabiana Lorencatto, Nicola McCleary, Justin Presseau, Geneviève Rouleau, Mina Tadrous, Simona C Minotti, Fahad Razak, Amol Verma, Laura Desveaux

**Affiliations:** 1 Institute for Better Health, Trillium Health Partners, 2085 Hurontario St, Mississauga, ON L5A-4G1 Canada; 2 Women’s College Hospital, Toronto, ON Canada; 3 Department of Medicine, University of Toronto, Toronto, ON Canada; 4 Department of Medicine, University Health Network, Toronto, ON Canada; 5 Program of Medicine, Trillium Health Partners, Mississauga, ON Canada; 6 School of Medicine, Wake Forest University, Winston-Salem, NC USA; 7 Li Ka Shing Knowledge Institute, St Michael’s Hospital, Toronto, ON Canada; 8 Interprofessional Practice Department, Unity Health Toronto, Toronto, ON Canada; 9 Lawrence Bloomberg Faculty of Nursing, University of Toronto, Toronto, ON Canada; 10 Institute for Health Policy, Management, and Evaluation, University of Toronto, Toronto, ON Canada; 11 University College of London, London, England; 12 Child Health Evaluative Sciences Program, SickKids Research Institute, Toronto, ON Canada; 13 Methodological and Implementation Research, Ottawa Hospital Research Institute, Ottawa, ON Canada; 14 School of Epidemiology and Public Health, University of Ottawa, Ottawa, ON Canada; 15 Nursing Department, Université du Québec en Outaouais, Gatineau, QC Canada; 16 Division of Biostatistics, Dalla Lana School of Public Health, University of Toronto, Toronto, Canada; 17 Institut du Savoir Montfort, Ottawa, ON Canada; 18 Leslie Dan Faculty of Pharmacy, University of Toronto, Toronto, ON Canada; 19 Department of Medicine, St. Michael’s Hospital, Toronto, ON Canada

**Keywords:** Variation in care, Length of stay, Implementation science, Internal medicine

## Abstract

**Background & objectives:**

Length of Stay (LoS) is a critical quality metric and focus of improvement efforts in healthcare. Successfully managing LoS depends on understanding the drivers of variation amenable to change. This study aims to (1) characterize physician-level variation in LoS; (2) identify physician actions associated with LoS; and (3) explore the individual-, team-, and hospital-level factors influencing this variation to generate hypotheses for further study.

**Methods:**

This mixed-methods comparative case study approach examined six General Internal Medicine (GIM) departments in Toronto, Ontario. Physician-level variation in LoS was calculated using a random-intercept negative binomial regression model and sensitivity analysis. Semi-structured interviews and ethnographic observations were conducted and analyzed using the AACTT Framework (Action-Actor-Context-Target-Time), the Consolidated Framework for Implementation Research (CFIR), and the Theoretical Domains Frameworks (TDF). Hospitals with the lowest and highest physician-level variation in LoS were compared.

**Results:**

Physician-level variation in LoS ranged from 1.7 to 7.0%, which—though modest numerically—represents meaningful differences in physician decision-making not explained by patient complexity, and no significant hospital-level effect was observed. Qualitative analysis from 12 observations and 67 interviews (32 GIM physicians and residents, 35 nurses and other health professionals) identified eight discrete physician actions influencing LoS, along with five individual-level factors and five team- and hospital-level factors. The nature of these factors was different when comparing hospitals with the lowest and highest variation. Organizational culture and perceptions of the patient population shaped physician perceptions of their professional role, while GIM departmental culture, structural characteristics, and communication networks informed physician beliefs about team capabilities and consequences of action (or inaction).

**Conclusion:**

This study highlights the complex interplay between physician actions and factors influencing physician-level variation in LoS. Interventions that target physicians but do not attend to team and hospital factors are likely insufficient to achieve sustained improvements in LoS. Aligning individual-level feedback and environmental restructuring with organizational values and needs of the patient population may offer a more promising approach to sustained improvement.

**Supplementary Information:**

The online version contains supplementary material available at 10.1186/s12913-025-13304-5.

## Background

Inpatient Length of Stay (LoS) is often viewed as a proxy for efficient hospital operations [1], making it an area of strategic focus given cost and capacity pressures [2–5]. Prolonged LoS also heightens complication risk, including infections and falls [6]. Numerous quality improvement (QI) interventions, including new clinical care [7–10] programs, innovative staffing models [11, 12], and enhanced care coordination [13–17], have fallen short on aspirations to effectively manage LoS. One potential explanation is that intervention success depends on the interaction between physician-level factors (e.g. training, learning style, and self-efficacy) [18–22] and team- and hospital-level factors (e.g., team members, available resources, organizational culture, and population needs) [23–27], which have yet to be systematically explored.

Verma et al. found that LoS varies substantially across physicians [28], with another study reporting that differences in physician practice can account for up to 75% of observed variation in post-operative LoS [29], suggesting individuals are a necessary target for intervention. Physician-level variation is ubiquitous despite extensive efforts to address it [30–35], highlighting the need to better understand the factors influencing physician variation to identify potential targets for intervention [36–38]. In the absence of this, interventions to improve LoS will continue to lack clarity and specificity on who needs to do what differently [39, 40], leaving physician recipients aware of the challenge but unsure on how and when to modify their behaviour. Further, existing literature typically relies on health administrative data to describe physician variations in LOS [28, 41, 42], but this data fails to capture essential influences on physician practice like team culture or clinical reasoning.

Without identifying modifiable factors driving physician-level variation, interventions addressing LoS will likely remain ineffective. Implementation science, which bridges the gap between evidence-based interventions and real-world application [43–45], offers a systematic approach for exploring what drives variation in LoS and which are amenable to change. This study uses a comparative case study design, treating each hospital as a distinct case, to [1] characterize the degree of physician-level variation in LoS across six General Internal Medicine (GIM) departments; [2] identify the physician actions associated with LoS; and [3] explore the modifiable factors at the hospital, team and physician-level that qualitatively explain lower and higher levels of physician-level variation in LoS and accordingly generate hypotheses for further study. This study aims to advance evidence to better inform the design and implementation of QI interventions targeting in-patient LoS. Practically, it generates hypotheses for further study and provides insights into how hospitals might structure the care environment to reduce variability in LoS.

## Methods

This mixed-methods study used a convergent parallel design [46] and was conducted between April 2021 and April 2023. We followed a comparative case study approach [47], treating each hospital as a distinct case to empirically investigate factors that influence LoS across six GIM departments in the Greater Toronto Area in Ontario, Canada. Cases were selected as they were members of a hospital research collaborative (GEMINI) and reflected a mix of both academic and community settings. Quantitative data were modeled and interpreted within each hospital before cross-case aggregation. Qualitative data were analyzed and organized by case to facilitate cross-case comparisons of contextual drivers. While data were collected concurrently, we completed the qualitative analysis first to ensure the team was blinded to outcomes while constructing the case studies, followed by the case-specific quantitative analysis. While the results are presented by data type (i.e., quantitative and qualitative), each hospital-level case was constructed and analyzed using both data sources in an integrated manner to ensure a comprehensive understanding of within-site dynamics prior to cross-case comparison. We report details of our qualitative methods according to the Consolidated Criteria for Reporting Qualitative Research [48] (COREQ) (Please see Additional File 1). Approval for this study was obtained through the Women’s College Hospital Research Ethics Board and all participants provided informed consent.

### Study setting

GIM departments within six large hospitals participated in the study, including four academic hospitals and two community hospitals, whose GIM services have been previously described [28, 49]. GIM admissions are almost entirely unplanned and occur primarily via the emergency department. Patients are assigned to attending physicians ‘quasi-randomly’ by the on-call internal medicine resident or staff physician at the time of admission in the emergency department. As a result, differences in outcomes within a large hospital sample are attributable to physicians and their teams, rather than patient differences [28, 50].

### Quantitative analysis

#### Study cohort creation

We extracted hospital encounter data between January 1, 2019 and December 31, 2021 from GEMINI [49], a hospital research collaborative [51], including patient characteristics, admission characteristics, and patient outcome data (Supplementary File #1). We created a three-level patient-physician-hospital nested dataset to facilitate the mixed effects analysis. Following Sergeant, Saha [52], we only included hospitalization encounters of patients admitted through the emergency department to maintain the quasi-random nature of patient assignment [28, 50]. Patients were attributed to the Most Responsible Physician (MRP), the physician responsible for the greatest portion of the corresponding hospital length of stay as recoded in the Discharge Abstract Database [52, 53]. Patients were excluded if they had a LoS of more than 30 days, as longer stays often lead to multiple physician handoffs which creates difficulties in attributing patient cases to a single MRP [28, 52]. To avoid potential clustering effects at the patient level [54], we only included the first admission of patients with multiple admissions during the study period. Considering that physicians may work at multiple hospitals during the study period, we only included patients treated by MRPs at their primary practice location (defined by the greatest patient volume). To avoid the potential unstable estimation related to small sample sizes [55], we only included MRPs with at least 50 patients assigned to them at their primary practice location over the study period.

### Statistical analysis

We used negative binomial regression with random intercepts to explore the variation in LoS that could be attributed to hospitals and MRPs, respectively [52, 56, 57], where level 1 variables included patient and admission characteristics, level 2 denoted the MRP, and level 3 denoted the hospital. The null nested three-level model (i.e., with no patient characteristics) revealed a hospital intra-class correlation coefficient (ICC) of 0.007, indicating no meaningful hospital-level effect. Subsequent analyses proceeded with two-level random-intercept negative binomial regressions, with hospital treated as a patient-level fixed effect when necessary. All models were fit with restricted maximum likelihood estimation.

We calculated the physician-level ICC to quantify the proportion of variance in LOS that may be attributable to MRPs. We adopted a staged modelling approach to investigate the ICC. First, we used the null model to estimate the unconditional physician-level ICC. Then, we included patient and admission characteristics to estimate the conditional ICC. To examine between-hospital heterogeneity, we replicated the null and full models for each individual hospital following Kirubarajan, Shin [57]. To visualize physician-level variations within each hospital, we plotted random intercepts that denote physician-specific deviations from a hypothetical average physician at that hospital after adjustment for level 1 variables, with 95% confidence intervals calculated using the conditional standard deviation.

We also calculated risk-standardized LOS ratios for each physician following Mohammed, et al. [58], examining the total number of predicted inpatient hours for a given MRP compared to the expected number of inpatient hours if a hypothetical average MRP had treated those patents. All analyses were performed in R version 4.1.3 [59] using or adapting code from the glmmTMB [60], performance [61], boot [62], and merTools [63] packages.

## Qualitative analyses

### Recruitment

The study physician lead at each hospital emailed all GIM staff about the study. Eligible interview participants included GIM physicians, medical residents (including chief residents), nurses (nurse practitioners, registered nurses, registered practical nurses), pharmacists, other health professionals (e.g., physiotherapists, occupational therapists, speech-language pathologists, discharge planners, care transition facilitators), and unit managers. Interested staff were advised to contact the study coordinator (DS) if they were interested in participating. Interested participants were provided with a study information sheet and verbal consent checklist upon contacting the coordinator. We targeted 10 interviews (split evenly between physicians and health professionals) and 3–5 physician observations at each hospital, with even distribution across participating hospitals. Eligible observation participants included GIM attending physicians, as their behaviour was the core focus of this study. All participants provided consent either verbally or through REDCap prior to interviews and observations.

### Data collection

Participants completed virtual interviews with a research associate (DS, M.Sc.). Basic demographic information (e.g., race/ethnicity, gender, age, years in practice, hospital, clinical role) was collected at the end of interview. A semi-structured interview guide (Supplementary Files #4 and #5) informed by the Theoretical Domains Framework [64] (TDF), the Consolidated Framework for Implementation Research (CFIR) [65], and structuration theory [66], was developed specific to this study to explore participant perceptions on the beliefs, attitudes, and perceived physician actions that influence patient LoS. The TDF is an individual-level determinant framework that assesses influences on individual behaviour [67], while the CFIR is one of the most commonly used determinant frameworks to assess contextual factors [65]. Structuration theory is a social theory about the creation and reproduction of social systems based on analyzing both the structure and its agents, without giving primacy to either [66].

The interview guide explored three topics: (1) organizational culture and team functioning, including individual roles and responsibilities, how care is coordinated, what information is shared and with whom, and how these factors influence the quality of care; (2) actions, decision-making, and teamwork as it relates to the care of a typical GIM patient represented via a case vignette; and (3) participant perceptions around the factors that influence LoS. All interviews were audio-recorded, transcribed, and anonymized by an independent third party.

In-person observations of physician participants were conducted by research staff (DS, KW, JV), guided by an ethnographic observation checklist (Supplementary File #6). Observations spanned a single shift of the physician (6–8 h) and focused on the attending physicians’ daily activities while on providing care on the in-patient unit, including all meetings and team interactions that occurred throughout the course of their shift. Research staff took extensive field notes, both handwritten and audio recorded, noting the nature of interactions, what and how information was shared, and who was present. Audio recordings were transcribed verbatim.

### Data analysis

Interview and observational data were analyzed using a template analysis [68], whereby TDF and CFIR domains were applied as a priori codes and the remaining data was coded inductively. Codes were generated at the team-level (i.e., GIM Department) across hospitals to support a comparative case study approach using the framework method [69]. A member of the research team (DS) independently coded two interview transcripts to develop a preliminary codebook with TDF and CFIR domains. The research team then met to discuss the codebook and review emerging inductive themes under the guidance of the principal investigator (LD). Interview transcripts were then divided up for coding using NVivo 14 (Lumivero), with each transcript being independently double-coded. The research team met to discuss coding and emergent themes on a hospital-by-hospital basis to construct case profiles at the hospital level. Observational data were used to validate interview themes and provide additional context to refine case (hospital level) understanding. These meetings were also used as an opportunity to review the analytic approach, resolve discrepancies, and ensure consistency in the coding approach across hospitals. To identify physician-level decisions and drivers of LoS, interview and observation data were organized by the Actor, Action, Context, Target, Time (AACTT) [70] framework to determine which discrete actions contributed to LoS in GIM, in what context(s), and who is involved. These were subsequently mapped to the corresponding TDF and CFIR domains to identify individual, team and organizational drivers of physician actions. Structuration theory was used as a sensitizing framework to ensure that TDF and CFIR drivers were considered equally and that primacy was not given to either [66]. Specifically, as data was coded to both TDF and CFIR domains where appropriate (referred to as the *intersection* of behavioural and contextual determinants), the authors constructed a matrix to simultaneously explore whether and how context interacted with individual behavioural determinants to impact physician-level actions. Multiple peer debriefing meetings were conducted, and results were refined in partnership with team members with qualitative expertise (LD, NI, FL, SAB) and validated with clinical hospital leads (AV, FR, LLS, AW, TT).

### Triangulation

To identify the drivers contributing to greater degrees of physician-level variation, we identified the two hospitals with the lowest levels of physician-level physician variation and the two hospitals with the highest, and conducted cross-case comparisons to generate hypotheses about what contributes to higher levels of physician-level variation in LoS.

## Results

### Objective 1: Understanding the degree of physician-level variation in length of stay

Our dataset included 44,371 individual patients seen by one of 204 GIM physicians across the six hospitals. The difference between the 10th and 90th percentile physician in average LoS ranged from 50 h at Hospital C to 108 h at Hospital F (see Table 1). In the two-level random-intercept negative binomial regressions, physician-level variation accounted for 7.4% of the total variation in LoS (i.e., ICC) in the null model, ranging from 3.9 to 11.4% across hospitals. Physician-level variation remained at 5.1% after adjustment for patient characteristics, ranging from 2.4 to 9.7% across hospitals and reflecting variation across a large cohort of patients from mild to severe illness. Although the absolute range of variation appears small, it is consistent with prior literature on physician-level effects and reflects systematic, non-random influences on LoS attributable to physician practice.

**Table 1:** Understanding physician-level variation in length of stay using two-level regression

		**All Hospitals**	**Hospital A**	**Hospital B**	**Hospital C**	**Hospital D**	**Hospital E**	**Hospital F**
**# of MRPs**		204	23	27	26	42	49	37
**# of patients**		44,371	7,232	6,132	5,784	7,413	7,372	10,438
**LOS in hours, median (Q1, Q3)**		104 (50, 196)	95 (45, 188)	108 (54,208)	106 (54, 193)	93 (46, 188)	113 (60, 209)	104 (49, 194)
Difference in average LoS between the 10^th^ and 90^th^ percentile MRP^a^		81	95	76	50	72	76	108
**Full Model**^b^	**MRP ICC**	0.051	0.097	0.0420	0.040	0.024	0.045	0.072

^a^The values were calculated by aggregating patient-level LoS to each MRP then ranking MRPs by their average LoS

^b^The full modeladjusted for fixed effects of age, gender, Charlson comorbidity index score, mLAPS, most responsible diagnoses, admission year, admission day, admission time, and the admitting hospital. For the individual hospitals, the full model included all covariates except the admitting hospital

**Fig. 1:**
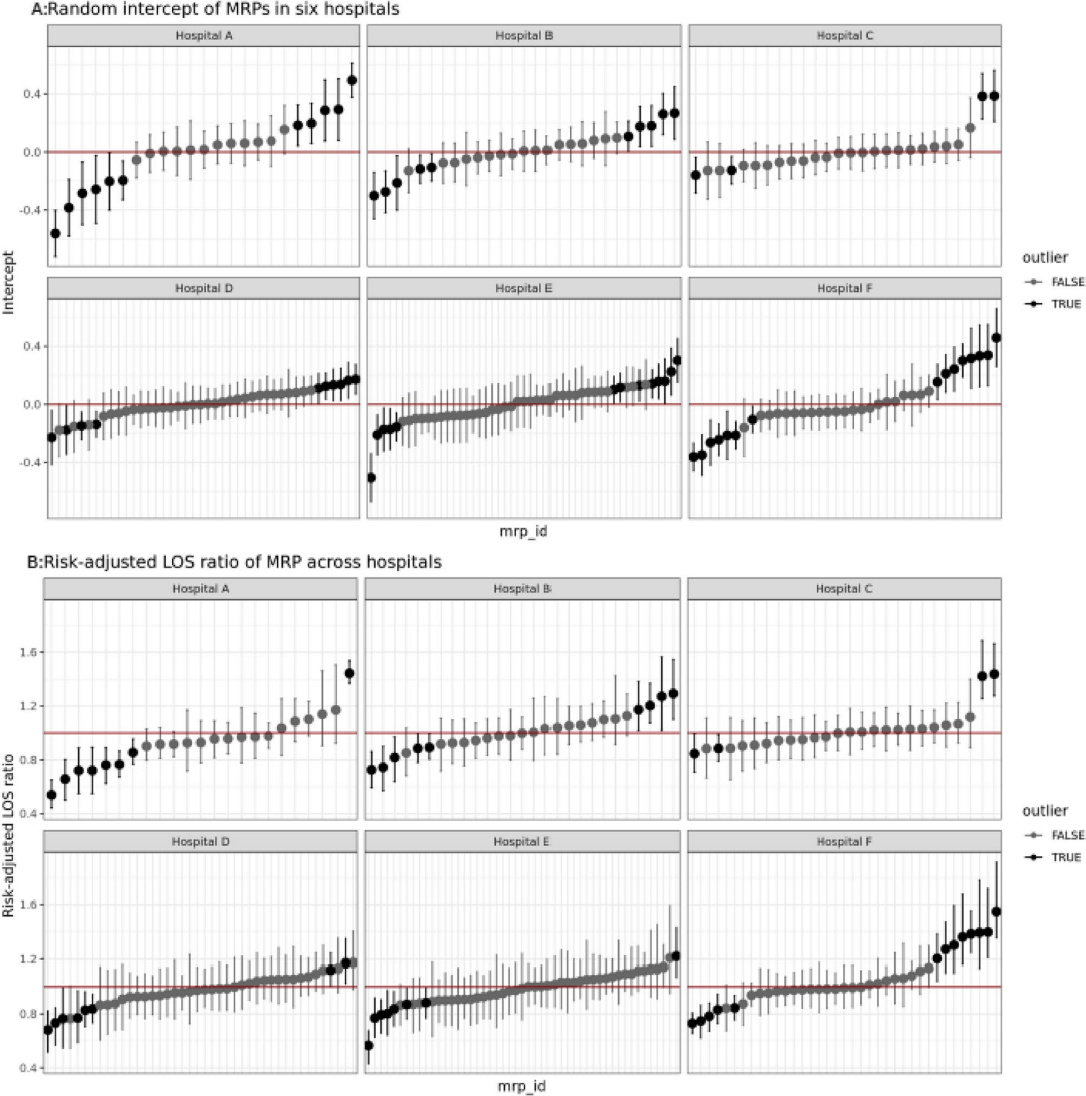
Physician-level variations in length of stay. **A** Physician-level estimates are random intercepts of the full model fit for each hospital. The horizontal line at 0 represents the hypothetical average physician within that hospital after adjustment for level 1 variables. Error bars represent 95% confidence intervals for each individual MRP, and black shading represents MRPs with significantly higher or lower estimates than hospital average. **B **Physician-level estimates are risk-adjusted LoS ratios of the full model. The numerator is the sum of predicted LOS values for a given MRP, considering their physician-specific random effect. The denominator is the sum of expected LoS values without physician-specific random effects. A ratio of 1 indicates that the MRP’s LoS aligns with what would be expected based on the case mix of their patients. The error bars represent 95% coverage intervals derived from cluster bootstrap methods. Black shading represents MRPs with significantly higher or lower estimates than expected

Meaningful physician-level variations in LoS were observed across all 6 hospitals (see Fig. 1 for a demonstration of within- and between-hospital variability in LoS). Variations were larger in some hospitals than others. For example, close to half of MRPs in Hospitals A and F (47.8% and 40.5%, respectively) had an average LoS that was significantly different than the hospital average (see Fig. 1 A). In contrast, Hospitals C and D had 15.4% and 23.8% of MRPs with an average LoS that differed significantly from their physician colleagues. Relative degree of physician-level variability is further supported in Fig. 1B, which displays physician-level risk-standardized LoS ratios by hospital in comparison to the expected LoS for a given MRP.

### Objective 2: Identifying discrete physician behaviours related to length of stay through the AACTT framework

Our qualitative dataset included a total of 67 semi-structured interviews with general internists (*n* = 27), medical residents (*n* = 5), nurses (*n* = 13), and other health disciplines (*n* = 22) across the six hospitals. Of the 67 participants, 43 (64%) were female and 24 (36%) were male (see Supplementary File #2 for participant demographics). Physicians ranged in age from 30 to 58 years (mean = 40 years, *n* = 20), with 1–28 years of practice experience (mean = 10 years, *n* = 20). Interdisciplinary staff ranged in age from 25 to 53 years (mean = 36 years, *n* = 34), with 1–25 years of practice experience (mean = 9.5 years, *n* = 33). Interviews ranged from 40 to 65 min (mean = 49 min). A total of 13 in-person observations were conducted across five of the six hospitals, for a total of 90 observed hours.

Qualitative interview participants identified discrete physician actions that influenced LoS, inclusive of activities from admission to discharge, which were validated through participant observations. The eight actions were common to GIM physicians across all six sites (see Table 2 for actions organized by the AACTT [70] framework). Of these, seven of the eight actions involved communication or collaboration with others, including the interdisciplinary care team or the patient and their caregiver(s).

**Table 2:** Discrete physician actions that impact patient length of stay

**#**	**Action**	**Actor**	**Context**	**Target**	**Time**	**Source**
	*Specify the behaviour in terms that can be observed or measured*	*Specify each person/people that does or could do each of the actions targeted*	*Specify the physical location, emotional context, social setting in which action is performed*	*Specify the person/people with/for whom the action is performed*	*Specify when the action is performed (the time/date/frequency)*	*Specify whether the behaviour was described during interview or observed in-person *
1	**Collaborate to establish goals of patient admission**	MRP, Junior and Senior Residents, Medical students	In person:1. Medical team rounds2. Interprofessional rounds3. EMR system	1. Members of the medical team^1^2. Members of the Interdisciplinary team^2^	1. Over the course of the care episode	1. Interviews2. Observations
2	**Discuss diagnosis and treatment plan with patient and caregivers**	MRP, Junior and Senior Residents, Medical students	In person:1. Patient bedside	Patient and patient caregivers	1. Over the course of the care episode	1. Interviews2. Observations
3	**Discuss roles and responsibilities with care team in relation to goals of admission **	MRP, Junior and Senior Residents, Medical students, charge nurses	In person:1. Medical team rounds2. Interprofessional roundsVirtual platforms:1. Email2. Phone calls3. Text messaging4. EMR’s instant message platform	1. Members of the medical team^1^2. Members of the Interdisciplinary team^2^	1. Over the course of the patient care episode	1. Interviews
4	**Establish & communicate criteria for discharge to the care team**	MRPor team member as proxy actor under direction of MRP (e.g., discharge coordinators, care transition facilitators)	In person:1. Medical team rounds2. Interprofessional roundsVirtual:1. Email2. Phone calls3. Text messaging4. EMR’s instant message platform	1. Members of the medical team^1^2. Members of the Interdisciplinary team^2^	1. In preparation for discharge	1. Interviews2. Observations
5	**Order investigations & procedures deemed necessary for discharge**	MRP or team member as proxy actor under direction of MRP (e.g., imaging coordinators, charge nurse, or resident)	In person: 1. Phone calls to the imaging department Virtual: 1. EMR system2. Internal requisition process	1. Imaging department2. Pathology department?	1. Over the course of the patient care episode	1. Interviews2. Observations
6	**Consult interdisciplinary staff for discharge evaluation and sign off**	MRP, Junior and Senior Residents, Medical students	In person:1. Medical team rounds2. Interprofessional roundsVirtual:1. Email2. Phone calls3. Text messaging4. EMR’s instant message platform	1. Members of the Interdisciplinary team^2^	1. In preparation for discharge	1. Interviews2. Observations
7	**Request necessary post-discharge services and supports **	MRP, Junior and Senior Residents, Medical students, charge nurses	In person:1. Medical team rounds2. Interprofessional roundsVirtual:1. Email2. Phone calls3. Text messaging4. Internal requisition process5. EMR’s instant message platform6. Fax	1. Members of the Interdisciplinary team^2^2. Internal, external and community health providers^3^	1. In preparation for discharge	1. Interviews2. Observations
8	**Discuss discharge plan with patients and caregivers**	MRP, Junior and Senior Residents, Medical students	In person:1. Patient bedside2. Family meeting (may be virtual)	1. Patient and patient caregivers2. Members of the Interdisciplinary team^2^3. External and community health providers^3^	1. In preparation for discharge	1. Interviews2. Observations

*MRP* Most Responsible Physician

^1^Medical team members include internal medicine physicians, clerks, and medical residents on GIM rotation

^2^Other health disciplines include Nurses, Physiotherapist, Occupational Therapist, Speech Language Pathologist, Pharmacist, Nutritionist, Patient Care Coordinator, Social Worker, Discharge Coordinator, Care Transition Facilitators

^3^Including but not limited to specialists, family physician, pharmacy, nursing home facilities, and rehabilitation facilities

Each action could be performed by the attending GIM physician or their assigned delegate, which varied across actions (e.g., senior or junior residents, medical students, discharge coordinators, etc.). These actions occurred across twelve different contexts (i.e., the physical location or social setting in which action is performed), with seven different targets (i.e., the person or people with/or for whom the action is performed), highlighting the variability in how and with whom these actions were enacted. In addition, while these actions occurred across all care interactions, their timing (i.e., when they occurred during the care episode) was variable.

### Objective 3: Identifying the drivers of physician-level variation

**Fig. 2:**
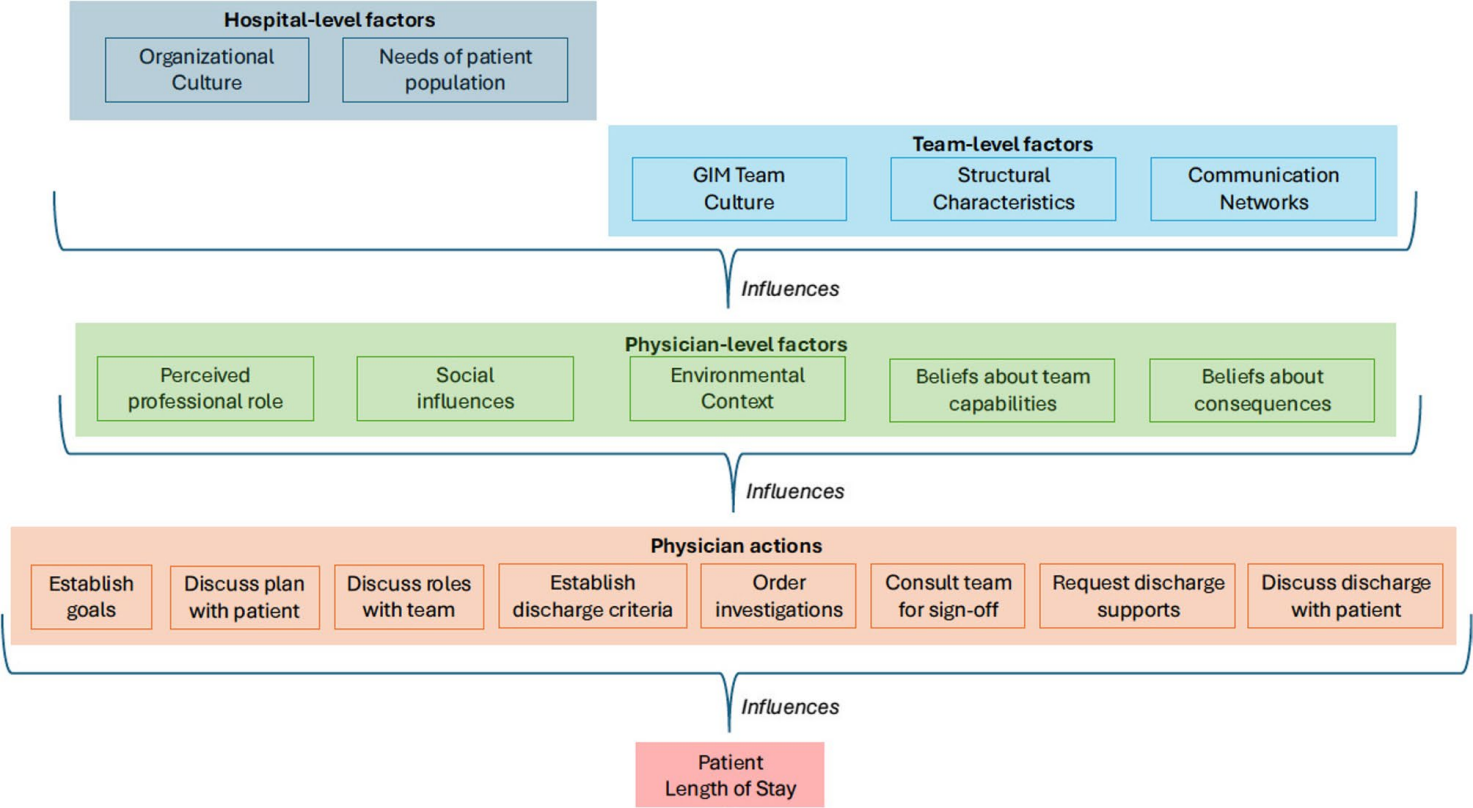
Multi-level factors influencing patient length of stay in GIM. Note: The relationships in this image are simplified for the purposes of illustration. A nuanced description of the intersection between factors is described in the text

Similar drivers (TDF and CFIR domains) were identified across the eight physician actions, irrespective of hospital (see Fig. 2, and Supplementary File #3 for TDF domains across actions). Physician actions were influenced by factors operating at the hospital- and team-level, including perceived patient needs and resources; the hospital’s organizational culture; the GIM team culture; communication networks within GIM; and the structural characteristics of GIM care. These hospital- and team-level factors influenced physician-level factors, including perceptions of their professional role; goals; sources of social influence; available resources; beliefs about team capabilities; and beliefs about consequences.

Informed by structuration theory, our analysis revealed that TDF and CFIR domains were not mutually exclusive, but that hospital- and team-level factors were influencing physician-level factors. Specifically, hospital organizational culture (CFIR) and patient needs and resources (CFIR) interacted to influence how physicians perceived their professional role and their related goals (TDF). At the team-level, GIM culture (CFIR), structural characteristics (CFIR), and communication networks (CFIR) influenced physicians’ beliefs about team member capabilities (TDF) and consequences of action (or inaction) (TDF). These intersecting factors provide both a lens through which variation at the physician-level can be preliminarily understood and a framework to understand what distinguishing higher variation sites from lower variation sites (refer to Table 3).

**Table 3:** Cross-case ccomparison of multi-level factors that influence length of stay across hospitals

		**Lower physician-level variability**		**Higher physician-level variability**	
		**Hospital D** MRP ICC 1.7%	**Hospital C** MRP ICC 2.8%	**Hospital F** MRP ICC 5.2%	**Hospital A** MRP ICC 7.0%
Organizational Level Factors	II. OUTER SETTINGPatient Needs & ResourcesThe extent to which the unique patient population is accurately understood and prioritized by the organization and influences actors within the organization.	Diverse patient population with limited access to healthcare resources, requiring additional coordination in complex cases	Patient population has a high degree of social and medical complexity and marginalization, requiring high degree of care coordination	Large subset of aging, co-morbid patients with moderate to high access to healthcare resources	Large subset of highly complex, co-morbid patients, many with high access to healthcare resources
	III. INNER SETTINGCulture (Organization)Norms, values and beliefs of medical teams.	Strong supportive cultureValues patient flow and mindful of healthcare system pressures	Supportive cultureValues comprehensive approach to care that acknowledges social and medical complexity Efficiency valued to the extent that it does not compromise individual care	Strong organizational culture of care quality and safetyValues continuous improvement toward discharge efficiency	Strong organizational culture of high performance and efficiencyValues patient flow and training clinicians
GIM Team Level Factors	III. INNER SETTINGCulture (GIM)Norms, values and beliefs of medical teams in relation to the inclusion of ID teams.	Interdisciplinary team highly valued; strong collaboration interdisciplinary staff can self-refer their services	Interdisciplinary team highly valued for complementary expertise; strong and equal collaboration	ID team valued but MRP drives which team members are engaged in care; degree of inclusion based on MRP priorities, but ID staff can advocate for their inclusion in the care plan	Interdisciplinary team valued; degree of inclusion based on MRP priorities
	III. INNER SETTINGNetworks & Communications (GIM)Structure and flow of information (e.g., in person meetings, EMR, etc.), both formal and informal, that determines and impacts relationship between MRP, medical, and ID teams.	Primary communication is virtual via the EMR; unstructured and variable face-to-face interactions; inconsistent rounding	Highly consistent morning rounding; high emphasis on face-to-face with variable use of formal and informal communication modalities	Highly consistent morning rounding: variable use of formal and informal communication modalities based on MRP and ID preferences	Consistent morning rounding with variable representation based on team lead; variable modalities and nature of additional communication based on MRP preferences
	III. INNER SETTINGStructural Characteristics (GIM)Delineated discharge supports available at the hospital	Unit-led discharge coordination	Team-based discharge coordinator	Central discharge coordinator shared across teams	Central discharge coordinator shared across teams

*EMR* Electronic Medical Record, *GIM* General Internal Medicine, *ICC* intraclass correlation coefficient, *ID* interdisciplinary, *MRP* Most Responsible Physician

### Drivers of physician-level variation among hospitals with low physician variability

Hospitals C and D had the lowest degree of physician-level variation (2.8% and 1.7% MRP ICC, respectively). Hospital D also had the lowest median LoS at 93 h while Hospital C had a median LoS of 106 h. Physicians at these hospitals had fairly homogenous self-described practice style compared to physicians at other hospitals, that were driven by the degree of standardization facilitated by their departmental and hospital context (refer to Table 3). Hospital culture and team processes and norms (CFIR) influenced individual behavioral determinants (TDF) and this interaction between domains explained nuances underlying how physician actions were carried out across hospitals (refer to Table 4).

**Table 4:** Intersection of TDF and CFIR constructs across hospitals with low and high physician-level variation

		Theoretical Domains Framework (TDF) Constructs				
		Professional Role & Goals	Social Influences	Environmental Context & Resources	Beliefs about Capabilities	Beliefs about Consequences
Hospitals with low physician level variation (Hospital C and D)						
**Consolidated Framework for Implementation Research (CFIR) Constructs**	**II. OUTER SETTING** **Patient Needs & Resources** The extent to which the unique patient population is accurately understood and prioritized by the organization and influences actors within the organization.	MRPs at both hospitals describe shared mindset of a holistic approach to patient care where their goal was to provide the medical expertise and work closely with the interdisciplinary teams. MRPs at Hospital D placed high emphasis on patient flow, while those at Hosp C engaged in teaching and mentoring learners	Both hospitals described patients and caregivers as strong social influences, whether advocating for a longer or shorter stay Hospital D also faced pressures from unit managers and hospital census load data	Staff at both hospitals demonstrated a high awareness of the relationship between the hospital and community resources to serve unique patient needs.		
	**III. INNER SETTING** **Culture (Organization)** Norms, values and beliefs of medical teams.					
	**III. INNER SETTING** **Culture (Medical and ID teams in GIM)** Norms, values and beliefs of medical teams in relation to the inclusion of ID teams.		GIM staff describe a positive dynamic between the broader organization and GIM; GIM staff felt supported by the organization.	MRPs and medical teams focused on ensuring medical care delivery, while ID and unit teams manage the overall care episode.		
	**III. INNER SETTING** **Networks & Communications ** Structure and flow of information (e.g., in person meetings, EMR, etc.), both formal and informal, that determines and impacts relationship between MRP, medical, and ID teams.					GIM teams believed that communication and information gaps during handover or a lack of clear roles and responsibilities led to delays in discharge.
	**III. INNER SETTING** **Structural Characteristics** Delineated discharge supports available at the hospital.			MRPs had few internal resources to rely on and work hard to connect patients to the necessary community resources. MRPs valued the support of team members who facilitated discharge (discharge planners at Hospital C and the charge nurse at Hospital D).		GIM teams believed that a lack of intentional and early planning for the patient’s care transition led to delays in discharge
	**III. INNER SETTING** **Structural Characteristics** Infrastructure components that support functional performance of the Inner Setting.			Hospital D: Communication was predominantly via the EMR system, with some unstructured face-to-face interactions. Structural adjustments made to support collaboration.		
**Hospitals with high physician level variation (Hospitals A and F)**						
**Consolidated Framework for Implementation Research (CFIR) Constructs**	**II. OUTER SETTING** **Patient Needs & Resources** The extent to which the unique patient population is accurately understood and prioritized by the organization and influences actors within the organization.	MRPs at both hospitals perceived their role to balance patient flow with high quality care, alongside leadership and teaching roles.	MRPs demonstrated variability in discharge thresholds and often sought to meet specific clinical criteria (rather than pursue a comprehensive set of discharge goals)			
	**III. INNER SETTING** **Culture (Organization)** Norms, values and beliefs of medical teams.		MRPs balanced multiple roles and were strongly influenced by census loads and organizational pressures toward efficiency, integrating patient and family preferences when feasible.			
	**III. INNER SETTING** **Culture (Medical and ID teams in GIM)** Norms, values and beliefs of medical teams in relation to the inclusion of ID teams.	MRPs oversee the entire care episode and sets team goals based on their individual perceptions of what needs to be prioritized.		GIM staff describe being disconnected and deprioritized in the context of their organizations’ Culture, morale, and motivation is determined more by the GIM teams, rather than by the hospital leaders.	MRPs variably engaged interdisciplinary teams based on perceived patient need.	
	**III. INNER SETTING** **Networks & Communications ** Structure and flow of information (e.g., in person meetings, EMR, etc.), both formal and informal, that determines and impacts relationship between MRP, medical, and ID teams.					MRPs believed that establishing and communicating goals of care drives discharge efficiency, and that timely communication had a significant impact on LoS.
	**III. INNER SETTING** **Structural Characteristics** Delineated discharge supports available at the hospital.				MRPs regularly relied on the internal post discharge units and often personally facilitated patients care transition and bridged internal and external care resources	
	**III. INNER SETTING** **Structural Characteristics** Infrastructure components that support functional performance of the Inner Setting.				Hospital A: Communication is predominantly in person, with a range of other secondary modalities used. The EMR system was less regularly used due to frequent technical challenges.	

*EMR *Electronic Medical Record,* GIM *General Internal Medicine*, ICC *intraclass correlation coefficient,* ID *interdisciplinary,* MRP Most Responsible Physician*

### Hospital organizational culture (CFIR) and patient needs and resources (CFIR) interacted to inform how physicians perceived their professional role and set their goals (TDF)

Hospital organizational culture and perceived patient needs and resources influenced physician perceptions of their professional role and their corresponding goals as a GIM physician. Physicians in both Hospital C and D described a strong, supportive organizational culture, bolstered by strong administrative and clinical leadership who were respected sources of support for the GIM department. Both hospitals described how the organization set a positive tone and provided a clear operating structure, creating an environment that balanced physician autonomy with a standardized way of providing care (thereby decreasing variability across physicians).

*“From the hospital*,* … value would be … in terms of effective communication within the team*,* working in a team environment to try actually achieve optimal care for patients because we all*,* in our culture*,* we all aim for improved patient outcome and to improve quality of care that we give to patients.”* Hospital C, ID3.

These collaborative cultures supported caring for patient populations with a complex interplay of social determinants of health that required a high degree of coordination within teams and between internal and external resources. While both hospitals described a range of resource challenges, most physicians in Hospitals C and D highlighted that the at-home reality and preferences of their patients directly influenced their decision-making. Teams were highly focused on ensuring all the right post discharge supports and safety nets were in place before discharge.

At Hospital C, physicians described the organization’s focus on caring for the whole person, motivating the team to plan toward comprehensive care upon admission and meet criteria for a safe patient discharge. Efficiency was focused on as an effort to serve the broader community and responsibly use public resources. In practice, this meant that physicians were comfortable ordering investigations, to ensure they could identify and address incidental findings before discharge – a reality of practice that was described as a key driver of variation at Hospital C that would contribute to a marginally longer LoS. Physicians at Hospital C described consistently engaging nurses and other health professionals as part of their professional role and strived to ensure access to post discharge supports and resources.

In contrast, physicians at Hospital D describe the organization’s primary focus on patient flow, where the diagnosis upon admission determined goals of admission and discharge. This was prioritized with the “collective in mind” (referring to the broader community and region they served) while striving to ensure adequate access community supports post discharge. Physicians paid close attention to hospital census, often engaging in communications via email or in person around capacity pressures. This influenced approaches to discharge, whereby physicians might plan for swift discharge during periods of higher pressure. While balancing capacity pressures, physicians at Hospital D also engaged consistently with patients and caregivers to discuss their care progress and post discharge plan and would sometimes agree to a longer LoS if requested by the patient, their caregivers, or the interdisciplinary team. Wherever possible, physicians leveraged the support of available post discharge clinics and once the patient was medically stable, preferred to arrange for additional investigations as follow up.

*“My personal style of practice is*,* at the time of admission*,* I tell the patient and the family the goals for their hospitalization. I also tell them the risks of being in hospital*,* including all the potential risks that they have by virtue of just being in a hospitalized environment. And I probably put a little bit of balanced fear in them*,* and every day when I round*,* they are asking for discharge if they’ve met all of the goals.”* Hospital D, P1.

### GIM culture (CFIR), structural characteristics (CFIR), and communication networks (CFIR) influenced physician’s beliefs about team member capabilities (TDF)

While physicians at Hospitals C and D had significantly different approaches to interacting with their interdisciplinary colleagues, participants at both hospitals described a high level of respect and collaboration, supporting effective communication to assign and clearly delineated responsibilities related to the care goals. Participants at Hospital C highlighted challenges with feeling aligned on criteria for discharge owing to the variability in terms of what safe and effective discharge looks like for a patient, at a specific point in time. Inconsistent communications among physicians, patients, and members of the care team resulted in misunderstandings that lead to eventual delays for patient investigations required for discharge, transitions to oral medications, and the organization of home care supports. Communication challenges were described as a significant point of stress during the discharge process, given their self-described higher loads of complex patients that are socio-economically disadvantaged who require considerable post-discharge supports.

*“We will hold them back and sometimes I just have to explain*,* once you can explain to the team your*,* the reason to the madness*,* they kind of understand actually you know*,* this actually will be a bounce back for me tomorrow if I do send this patient home because we’ve seen it so many times. And as I said*,* I’ve been in this role now for 18 years. I have seen a lot. So*,* once you just explain to the doctors that the safest discharge for this person would be Monday where we know that all the supports will be in and ready for this patient.”* Hospital C, ID1.

Physicians at Hospital D described that their environment necessitated quick, clear, and streamlined communication between physicians and the unit-based patient care teams. Communication was predominantly via the electronic medical record (EMR) system, with some unstructured face-to-face interactions. The EMR system, and its embedded instant messaging module, was a key structural resource to enable collaboration at Hospital D, where the lack of geographic collocation of GIM physicians and patients presented a structural barrier to easy collaboration between the physician and interdisciplinary team.

*“[With the EMR chat feature] you can add multiple members to a chat [and] have a mini meeting on patients at times*,* if I’m including the nurse*,* the dietician*,* the doctor and kind of getting my update*,* and they give their respective updates. It allows us to all receive the same information instantaneously. –* Hospital D, ID2.

## Drivers of physician-level variation among hospitals with higher physician variability

Hospitals A and F had the highest levels of physician-level variation (7.0% and 5.2% MRP ICC, respectively), with corresponding reports from interdisciplinary staff that patient care is largely determined by physician preferences, in contrast to Hospitals C and D where physician practice styles were relatively more homogenous (refer to Table 3). The hospital culture and degree of standard departmental processes and norms (CFIR) resulted in less standardization, which led to greater influence of individual physician-level factors (TDF) determining how physician actions were carried out across hospitals (Table 4).

### Hospital organizational culture (CFIR) and patient needs and resources (CFIR) interacted to inform how physicians perceived their professional role and set their goals (TDF)

Both hospitals described serving a largely affluent, structurally advantaged patient population with greater access to resources and social supports. This resulted in a lower perceived burden on the care team to facilitate post discharge support and resources, creating flexibility for physicians at both hospitals to plan goals of admission and discharge criteria based on individual perception of current priorities. In contrast to Hospitals C and D, participants in Hospitals A and F described feeling disconnected and deprioritized in the context of their organizations. and participants at Hospital A described the environment as ‘GIM versus the hospital’. GIM culture, morale and motivation was driven by the GIM leaders and teams, more than the hospital leaders.

“… *despite the large footprint we have within the inpatient side of the hospital*,* we’re not a priority (…). There’re no flashy ad campaigns there’s no major donors*,* we just (…) grind it out and see the patients (…) despite all the attention (…) to other programs (…)”* Hospital F, P5.

***“****I think the organization’s pressure on GIM that the culture needs to based on efficiency kind of bleeds through. So*,* while people want to focus or understand that it would be wonderful if we’re focused on collaboration*,* communication*,* patient care*,* patient safety*,* there’s the overarching kind of cloud of*,* everything needs to move very quickly*,* everything needs to be as efficient as possible and we do not have time to do any of those things*,* move as fast as possible.”* Hospital A, ID5.

Hospital communications about and messaging around capacity and census load pressures directly influenced physician decision-making around discharge criteria at both hospitals. Many physicians in Hospital F paid close attention to hospital census, efficiency metrics, and Alternative Level of Care (ALC) bed availability which determined how they approached discharge. Physicians at Hospital A explained that the organization’s focus on efficiency and patient census created an overall culture of urgency and efficiency that demanded high performance. This overarching tone of high performance created an environment where physicians had to regularly evaluate and re-balance competing priorities against the backdrop of a culture of continuous improvement and teaching and mentorship of multiple learners. As a result, their primary focus as a GIM physician varied depending on timing and context. Physicians at Hospital A determined goals of admission (Action 1), division and delegation of tasks, and thresholds for discharge based on patient volumes, learner competence, and resource availability.

*“I think the only unique part at [Hospital A] is that because we’re so used to very high volumes*,* we*,* most of the attendings come in earlier*,* just anecdotally*,* than I think typically occurs at other hospitals. And as a consequence*,* the patients are only reviewed between the staff physician and the admitting resident that was on overnight. But by contrast*,* in other places I’ve worked the volumes of new admissions would often allow for the entire team [medical and interprofessional teams] to be present when the case is reviewed*,* which helps with sort of the handover.”* Hospital A>, P4.

Physicians at Hospital F similarly described competing priorities which manifested through managing the commitments of multiple formalized leadership roles in addition to clinical and teaching responsibilities, including those related to quality, research, and academic affairs. They described prioritizing system needs (e.g., organizational capacity pressures) while managing clinical care and integrating patient and family preferences when feasible while discussing discharge plans with patients and caregivers. Some physicians who felt confident in community and out-patient supports were described as more “aggressive” by their colleagues, with their propensity to discharge quickly described as a product of their ability to maintain continuity and follow-up with patients as needed. Others described readmissions as an unavoidable reality due to limited resources and capacity restraints.

*“Yeah*,* I think some physicians are not as aggressive*,* I think part of it is ability to follow patients. So*,* some clinicians are very happy to follow patients virtually or in their own clinics when they leave the hospital. Other physicians who might not have that type of practice or ability are probably more reluctant to send people home a day early*,* so to speak.”* Hospital F, P3.

### GIM team culture (CFIR), communication networks (CFIR), and structural characteristics (CFIR) led to varying physicians beliefs about team member capabilities (TDF) and consequences (TDF)

In contrast to the low variability hospitals (Hospitals C and D), there was more diversity in how physicians at Hospitals A and F described their engagement with interdisciplinary professionals. Physicians focused on consulting within medical team to achieve the clinical goals of care and may not consistently facilitate dialogue around roles and responsibilities of interdisciplinary staff in the patient care episode. In such cases, the interdisciplinary team often made assumptions about how to work best with the attending physician, either informed by prior experience or insights from colleagues. Inconsistency in whether and how well-informed members of the interdisciplinary team were of the attending physicians’ decision processes and desire for status updates also contributed to confusion. Beyond daily inter-professional rounds, Hospital A did not have standardized communication norms and communication frequency and modality (e.g., email, text messaging, EMR chat etc.) was based on physician preferences.

*“If [the physician says] that the patient was supposed to be discharged at the rounds and then like it’s 13:00 and then you’re like the patient’s still not discharged what’s happening? …. Now we have to page the team ourselves*,* “Hey were told by the in-charge during rounds that they were supposed to be discharged.” “Oh no –” and then they’ll say “No*,* oh no*,* you know*,* they have to get seen by this*,* you know*,* hepatology first or they have to do this test first” but like no one tells us anything. So we’re kind of like oh OK meanwhile we told the patient that they might be discharged today and then no one says anything afterwards. No one follows up with the patient and no one follows up with the nurse. It may be talked about within their own team*,* but it just doesn’t get relayed to us anymore.”****Hospital A***,*** ID4***.

## Discussion

This mixed-methods study applied implementation science to examine factors influencing quantified physician-level variation in LoS across six hospitals. Five key hospital- and team-level factors were identified: organizational culture; perceived needs of the patient population; team culture; the nature of collaboration with interdisciplinary teams; and the structure of roles and discharge resources. These factors shaped physician perceptions of their role, goals, beliefs about team member capabilities, and beliefs about the consequences of action. Hospitals with lower physician-level variation exhibited stronger team dynamics, characterized by a clear understanding of roles, trust in the capabilities of all team members, and consistent communication.

Previous qualitative research highlights organizational climate [71], a culture of shared beliefs and norms [26], and patient characteristics [72–74] as factors influencing physician decision-making. Our work builds on this research by highlighting the interplay across factors- a key element to support the design of effective improvement interventions [75, 76]. Our findings reinforce prior literature referencing strong and weak situations, where a strong situation provides clear cues for behaviour in a way that produces consistent actions in line with situational constraints [77–79]. The movement towards evidence-based care is based on the notion that cultivating strong contexts mitigates decisions based on a healthcare professional’s “unsystematic clinical experience” [80], which may manifest as physician-level variation in outcomes. While the absolute magnitude of variation in LoS may appear modest, our qualitative findings suggest that even small differences reflect meaningful variation in physician decision-making shaped by local context. This underscores a disconnect between what appears quantitatively minimal and what is experienced qualitatively as significant by care teams and patients.

Hospitals with relatively lower physician-level variation seemed to exhibit consistent collaboration between medical and interdisciplinary teams, though collaboration style varied across hospitals. This was characterized by a shared understanding of roles and responsibilities and supported by structured, team-based resources. Our results suggest that physician-level variation in LoS is largely influenced by hospital- and team-level factors [54], aligning with QI research that interventions should focus on the inter-connections between systems and individual actors [81, 82] and address two or more levels of influence simultaneously [76, 83]. This is also consistent with scholarship that suggests strong professional culture limits individual differences [77] and promotes consistency across all members of the team. Restructuring the environment in collaboration with teams effectively improves collaboration and guideline-based care [84], emphasizing the need for a team-based lens to improving LoS. Evidence based strategies may include training team members together, developing consistent practices toward information sharing and fostering an organizational culture through mechanisms that balance interdisciplinary and medical collaboration [85]. Future research should attend to the influence of context when piloting and evaluating interventions to inform what works best, for which teams, in which contexts.

Our mixed-methods approach provides insights into how participants perceive their actions and those of their team members impact patient LoS, supported by direct observations of physician behaviour and interactions. However, the Hawthorne effect may have influenced observed behaviour [86], thought this is mitigated through triangulating the interview and observational data. While our qualitative data is valuable for hypothesis generation, it does not establish directionality or causality. The identified factors likely interact bi-directionally and future research should test these relationships through methods like coincidence analysis to assess conditions (e.g., strong organizational culture, strong team collaboration, centralized discharge resources, etc.) coincident with less variability in LoS [87]. We are unable to quantitatively determine the degree to which the individual actions identified contribute to variation in LoS, however this work provides insight into the areas of influence where measurement is likely to be valuable. Despite qualitative findings on hospital-level factors, we did not detect meaningful hospital-level variation in LoS, likely due to the sample size (*n* = 6 hospitals). Future studies should explore 3-level models with a larger sample size that includes a minimum of 30 clusters [88]. While the composition of interdisciplinary care teams varied across the hospitals, examining the implications was beyond the scope of this study. Lastly, data collection during the COVID-19 pandemic may have also introduced recall and recency bias, despite efforts to prompt reflection on pre-pandemic experiences.

## Conclusions

As health administrators, clinical leaders, and professional organizations continue to design and deliver improvement interventions aimed at addressing capacity challenges, our study helps illuminate the interplay between hospital-, team-, and physician-level factors influencing the degree of physician-level variation in LoS. Improvement interventions targeting physician-level change may be low-yield and burdensome in the absence of a multi-level approach targeting structural changes. Specifically, interventions should target organizational culture to cultivate ‘strong situations’, where collective norms are encouraged by structures and processes; facilitate the effective flow of information between the various clinical care teams; and ensure clear roles and responsibilities across members of the care team. Aligning individual-level feedback and environmental restructuring with organizational values and patient population needs is a promising approach, as these factors interact to influence clinical decision-making and patient LoS.

## Supplementary Information

Supplementary Material 1

## Data Availability

The manuscript has analysed data included as electronic supplementary material. The raw datasets generated and analyzed during the current study are not publicly available as they are interview transcripts or observational field notes that might risk identify the participants. To maintain anonymity data files will be available from the corresponding author on reasonable request.

## Abbreviations

GIM: General Internal Medicine
AACTT Framework: Action-Actor-Context-Target-Time Framework
CFIR: Consolidated Framework for Implementation Research
TDF: Theoretical Domains Frameworks
LoS: Length of Stay
QI: Quality Intervention
MRP: Most Responsible Physician
ICC: Intra-class correlation coefficient
EMR: Electronic Medical Record
ALC: Alternative Level of Care
CIHR: Canadian Institute of Health Research
