# Supplementary material for: Applying an implementation science lens to understand physician-level variation in patient length of stay in internal medicine
Source: BMC Health Serv Res. 2025 Oct 3;25:1292. doi: 10.1186/s12913-025-13304-5 (PMC12495839; doi:10.1186/s12913-025-13304-5)
Supplement: Supplementary file 1 — Supplementary Material 1 [file 12913_2025_13304_MOESM1_ESM.docx]

**Supplemental File 1: Data dictionary**

| **Variable type** | **Variable** | **Definition** | **Categories/values** |
| --- | --- | --- | --- |
| **Outcome** | Length of stay | Difference in hours between discharge date and time and admission date and time, where admission date refers to the date and time when the patient was officially registered as an inpatient for the current hospital admission^1^. | Values range from 0 to 743 |
| **Hospital-level variable** | Admitting hospital | Sampled hospitals. | Hospital ID |
| **Physician-level variable** | Most responsible physicians (MRP) | The physician who was assigned to the patient when they were admitted from emergency department to GIM^2^ and who is considered most responsible for the care and management of the patient^3^. | MRP ID |
| **Patient-level variables** |  |  |  |
| 1. Patient characteristics | Age | Patient's age in years at the time of admission. | Values of 18 and over |
|  | Gender | The physical sex of the patient listed at the admitting hospital. | Female, Male |
|  | Most responsible discharge diagnosis | Determined by aggregating the ICD-10 code into clinically meaningful disease categories based on clinical classifications software^4^. | 11 categories, including the top 10 diagnoses and “others” (all remaining diagnoses) |
|  | Charlson Cormobidity Index at admission on both in-patient and ER diagnoses | A validated measure of categorizing comorbidities of patients based on the International Classification of Diseases (ICD) diagnosis codes^5^. | 0, 1, and 2+ |
|  | Modified Laboratory-based Acute Physiology Score at admission (mLAPS) | A validated measure of illness severity based on laboratory results, with a higher score indicating greater risk inpatient and 30-day mortality when combined with age, comorbidity, and sex^6 7^. | Values range from 0 to 231 |
| 1. Admission characteristics | Admission year | The year that the patient was officially registered as an inpatient for the current hospital admission. | 2019, 2020, and 2021 |
|  | Admission day | The day that the patient was officially registered as an inpatient for the current hospital admission. | Weekday, weekend |
|  | Admission time | The time that the patient was officially registered as an inpatient for the current hospital admission. | Daytime (08:00-17:00), night-time (otherwise) |

**Supplemental File 2: Participant Demographic Information**

| **Role** | **Interviews** | **Observations** |
| --- | --- | --- |
| Physicians | 27 | 13 |
| Resident | 5 | - |
| Nurse (incld administrative roles) | 14 | - |
| Other Health Professionals* | 21 | - |
| **Age** | **Interviews** | **Observations** |
| 20-29 | 10 | - |
| 30-39 | 18 | 7 |
| 40-49 | 8 | - |
| 50-60 | 5 | 3 |
| Undisclosed | 7 | 3 |
| **Gender** | **Interviews** | **Observations** |
| Female | 43 | 2 |
| Male | 24 | 11 |
| **Race/Ethnicity** | **Interviews** | **Observations** |
| African | 5 | - |
| Caucasian/ European | 34 | 7 |
| East Asian | 8 | 4 |
| Mixed Heritage | 4 | 1 |
| Middle Eastern | 2 | - |
| South Asian | 8 | 1 |
| South-East Asian | 6 | - |
| **Years in GIM** | **Interviews** | **Observations** |
| 0-5 | 27 | 5 |
| 6-10 | 14 | 3 |
| 11-15 | 6 | 5 |
| 16-20 | 7 |  |
| 21-25 | 4 |  |
| 25+ | 2 |  |
| Undisclosed | 8 |  |
| **Years in current role** | **Interviews** |  |
| 0-5 | 34 |  |
| 6-10 | 13 |  |
| 11-15 | 4 |  |
| 16-20 | 4 |  |
| 21-25 | 2 |  |
| 25+ | 2 |  |
| Undisclosed | 8 |  |

*Health professionals include physiotherapists, occupational therapists, speech language pathologists, pharmacists, nutritionists, patient care coordinators, social workers, discharge coordinators, and care transition coordinators.

**Supplemental File 3: TDF Domains associated with the 8 discrete physician actions that impact patient length of stay**

|  | **Professional role and identity** | **Goals** | **Environmental context and resources** | **Social influences** | **Beliefs about capabilities** | **Beliefs about consequences** |
| --- | --- | --- | --- | --- | --- | --- |
| **1. Collaborate to establish goals of patient admission** | **○** | **○** | **○** | **○** |  |  |
| **2. Discuss treatment and care plan with patient and caregivers** | **○** |  |  | **○** |  | **○** |
| **3. Discuss roles and responsibilities in relation to goals of admission with care team** | **○** |  | **○** |  | **○** |  |
| **4. Establish & communicate criteria for discharge to the care team** |  | **○** | **○** |  |  | **○** |
| **5. Order investigations & procedures required for discharge** |  | **○** | **○** | **○** |  | **○** |
| **6. Engage Interdisciplinary staff for discharge evaluation and sign off** |  |  | **○** |  | **○** | **○** |
| **7. Request necessary post-discharge services and supports** |  |  | **○** |  |  | **○** |
| **8. Discuss discharge plan with patient and caregivers** | **○** |  |  | **○** |  |  |

**Supplemental File 4: Qualitative Data Collection: Interview Guide for Physicians and Residents**

1. How is patient care organized in General Internal Medicine?
2. To what extent, if any, does how things are done on a patient's care team depend on what the **team** as a whole is supposed to accomplish?
3. To what extent, if any, does how things are done on a patient's care team depend on what **you** are supposed to accomplish?
   1. Prompt: What are the norms and values that dictate how you should function within your team?
4. What are the spoken or unspoken set of rules that govern how the team interacts with one another?
   1. Prompt: What are the organizational expectations around teamwork?
   2. Prompt: What organizational resources are available, if any, to build a sense of community within the team?
5. How would you describe communication across your medical team?
   1. Prompt: Can you describe how this is similar to, or different from your communication with your interprofessional/allied health colleagues?
   2. Prompt: What questions are asked, by whom, and what communication channels are used?
6. [Reflect back site-specific context obtained from responses to #1-5] You’ve mentioned X, Y, Z features of team functioning in GIM at your hospital. To what extent do these features influence your ability to provide quality patient care?
   1. Prompt: Please describe an example.
7. If you saw or believed someone on your team made what you perceived as a minor error, what would you do?
   1. Prompt: Describe the steps you would take and who you would communicate this to.
8. To what extent do you feel like you are able to identify or speak openly about minor errors in your own decision making?
   1. Prompt: Who do you usually go to for advice or feedback?

**Please consider the following case study to help us understand how decisions are being made.**

**Case: Consider a 50-year-old woman admitted with dysuria, flank pain and fever. She has frequent cystitis treated as an outpatient with oral antibiotics. A clinical diagnosis of pyelonephritis is made, and the patient is start on intravenous ceftriaxone. Urine cultures on day 2 grow E coli susceptible to ceftriaxone. She is afebrile at day 3. On Day 4 she is transitioned to amoxiclav. On Day 5 she is still fatigued, but the team is considering discharge home to complete a 14-day course of antibiotics.**

1. Consider the case above, what actions would you take to determine whether this patient should be discharged today versus extending their hospitalization?
   1. Prompt: Who else on the team would be involved in this decision and what is the degree of their involvement?
   2. GEMINI data suggests that for the typical GIM physician there is no relationship between their patients’ average LOS and 30-day readmission, indicating an opportunity to reduce LOS without harming patients (but this may not apply for both all patients and for all physicians). Does this change your decision-making regarding discharge?
2. From your perspective, what impacts LOS?
   1. Prompt: What do you have a control over as a physician? As a care team?
   2. Prompt: What is beyond your control?

**I would like to take a moment to reflect on the discussion we just had and explore whether your experience has shifted during/because of the COVID pandemic.**

1. To what extent has COVID impacted:
   1. The way you and your colleagues provide care?
   2. Your ability to provide quality care to your patients?
   3. Communication within your medical team? Communication with your interprofessional/allied health colleagues?
   4. The culture of teamwork among the professionals that care for your patients?
2. Before we conclude this interview, is there anything else you’d like to share about organizational culture and how and whether team functioning may impact variation in care?

**Supplemental File 5: Qualitative Data Collection: Interview Guide for Interdisciplinary Professionals**

1. How is patient care organized in General Internal Medicine?
2. To what extent, if any, does how things are done on a patient's care team depend on what the **team** as a whole is supposed to accomplish?
3. To what extent, if any, does how things are done on a patient's care team depend on what **you** are supposed to accomplish?
   1. Prompt: What are the norms and values that dictate how you should function within your team?
4. What are the spoken or unspoken set of rules that govern how the team interacts with one another?
   1. Prompt: What are the organizational expectations around teamwork?
   2. Prompt: What organizational resources are available, if any, to build a sense of community within the team?
5. How would you describe communication across your interprofessional/allied health colleagues?
   1. Can you describe how this is similar to, or different from your communication with the medical team?
   2. Prompt: What questions are asked, by whom, and what communication channels are used?
6. [Reflect back site-specific context obtained from responses to #1-5] You’ve mentioned X, Y, Z features of team functioning in GIM at your hospital. To what extent do these features influence your ability to provide quality patient care?
   1. Can you describe an example that highlights this?
7. If you saw someone on your team make what you perceived as a minor error when treating a patient, what would you do?
   1. Prompt: Describe the steps you would take and who you would communicate this to.
8. To what extent do you feel like you can speak openly about minor errors in your own decision making?
   1. Who do you usually go to for advice or feedback?
9. From your perspective, what impacts LoS
   1. *Follow up*: What do you have a control over as an individual? As a team?
   2. What is beyond your control?

**I would like to take a moment to reflect on the discussion we just had and explore whether your experience has shifted during/because of the COVID pandemic.**

1. To what extent has COVID impacted:
   1. The way you and your colleagues provide care?
   2. Your ability to provide quality care to your patients?
   3. Communication within your medical team? Communication with your interprofessional/allied health colleagues?
   4. The culture of teamwork among the professionals that care for your patients?
2. Before we conclude this interview, is there anything else you’d like to share about organizational culture and how and whether team functioning may impact variation in care?

**Supplemental File 6: Ethnographic Observational Checklist**

The purpose of this document is to assist the research team in conducting ethnographic observations. This document is meant as a guide only and should not be regarded as exhaustive or prescriptive. Researchers are encouraged to be note down anything they deem as relevant to the research objectives and to be descriptive rather than interpretative.

**Immediately prior to commencing the observation:** Before the observed clinician meeting, the lead of the meeting will remind participants that a member of the evaluation team is sitting in on the call to learn about the processes and how decisions are made within the program. The sessions will be recorded, and the research team member will be taking general notes about the process. The observer can be asked to leave at any time and anyone on the call can ask the observer to leave by sending them a private chat message on Zoom that will not be visible to the rest of the team. If one person objects to having the observer present, the observer will leave.

| Domains of observation | Notes |
| --- | --- |
| Verbal behaviours and interactions (*dynamics of interactions i.e., who speaks to whom and for how long, who initiates interaction, tone of voice)* |  |
| Physical behaviours and gestures (*what people do, who does what, who interacts with whom, who is not interacting)* |  |
| Personal space (*how close people stand to one another, what individuals’ preferences concerning personal space suggest about their relationships)* |  |
| Human traffic (*the number of people, people who enter, leave, and spend time at the observation site)* |  |
| Physical space (*the physical space of the observation site)* |  |
